# Bioprinting and Differentiation of Stem Cells

**DOI:** 10.3390/molecules21091188

**Published:** 2016-09-08

**Authors:** Scott A. Irvine, Subbu S. Venkatraman

**Affiliations:** School of Materials Science and Engineering, Nanyang Technological University, 50 Nanyang Avenue, Singapore 639798, Singapore; assubbu@ntu.edu.sg

**Keywords:** 3D bioprinting, stem cells, lineage commitment, differentiation, bioinks

## Abstract

The 3D bioprinting of stem cells directly into scaffolds offers great potential for the development of regenerative therapies; in particular for the fabrication of organ and tissue substitutes. For this to be achieved; the lineage fate of bioprinted stem cell must be controllable. Bioprinting can be neutral; allowing culture conditions to trigger differentiation or alternatively; the technique can be designed to be stimulatory. Such factors as the particular bioprinting technique; bioink polymers; polymer cross-linking mechanism; bioink additives; and mechanical properties are considered. In addition; it is discussed that the stimulation of stem cell differentiation by bioprinting may lead to the remodeling and modification of the scaffold over time matching the concept of 4D bioprinting. The ability to tune bioprinting properties as an approach to fabricate stem cell bearing scaffolds and to also harness the benefits of the cells multipotency is of considerable relevance to the field of biomaterials and bioengineering.

## 1. Introduction

Currently, the availability of transplantable organs does not meet demand. In the USA, 121,070 people require an organ transplant, however only 2553 were performed in 2015 and approximately 22 people die daily while waiting [1]. A solution to this shortfall is the fabrication of organs by tissue engineering. One technique that could potentially meet this demand is 3D bioprinting. Theoretically, this approach can generate an artificial organ of customized size and shape for patient-specific therapy [2,3].

Over recent years, 3D printing, also known as free from fabrication (FFF), rapid prototyping (RP) and additive manufacturing (AM) has become of great interest in the imagination of the general public. Research in biotechnology and bioengineering have also harnessed similar manufacturing techniques to generate solutions as both research tools and for the fabrication of biomedical scaffolds [4,5]. Bioprinting refers to the controlled delivery of biological materials both cellular and acellular. This article will focus on the former, and how it can be exploited to deliver stem cells whilst harnessing their multipotency.

**Figure 1:**
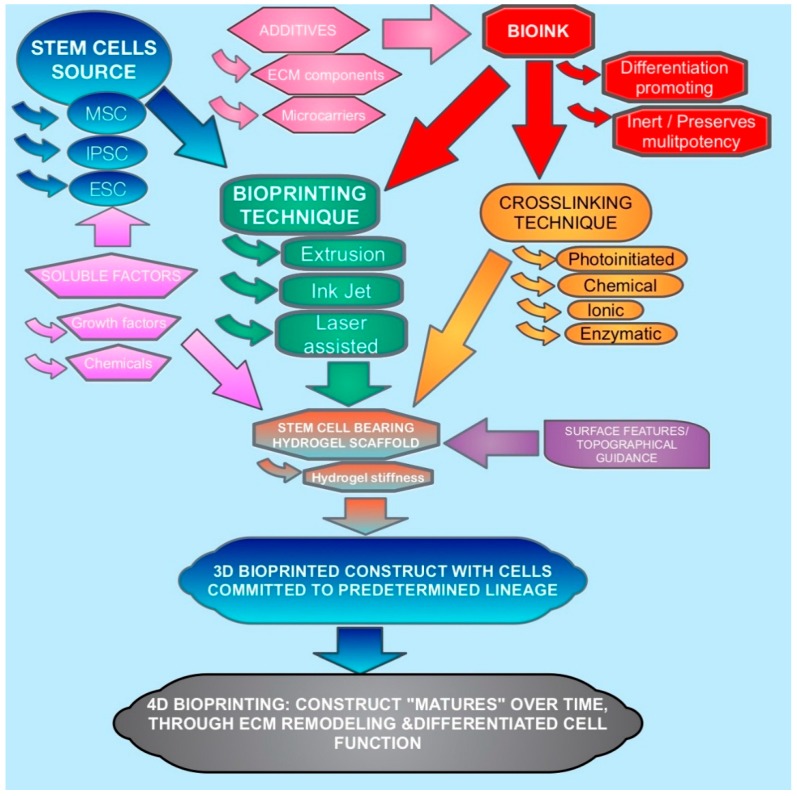
Diagram demonstrating interplay of the factors and variables involved in the 3D bioprinting and differentiation of stem cells as discussed in this article.

Fabrication with current 3D printing techniques require a printing material to be delivered in a fluid or powder, which then must rapidly set to maintain the integrity of the print. This, however, traditionally required a print material to be printed using high temperatures for both metals and thermosetting polymers, or to be dissolved in volatile solvents. Such techniques are suitable for the fabrication of acellular scaffolds for bioengineering. However, if cells can be included into a cytocompatible printing material and process, then the subsequent construct will be immediately cellularized with a consistent cell distribution throughout. Furthermore, the distribution of cells and different cell types can potentially be placed and orientated to more mimic the desired natural tissue [6,7]. The factors involved in the 3D bioprinting of stem cells and their interplay as discussed in this article are depicted in Figure 1.

Classical approaches towards predetermined lineage differentiation of stem cells in vitro have mainly focused on chemical and growth factors (i.e., soluble factors). However, with advances in bioengineering and biomaterials, it has been realized that other factors such as environmental and substrate properties can also stimulate the process [8]. As in vivo stem cell differentiation occurs under the influence of multiple stimuli in the stem cells’ microenvironment, a similar effect can be harnessed in bioengineering [9]. Bioprinting can involve several variables that predetermine lineage, such as soluble factors, additives and bioink choice (Figure 1). Other bioprinting components of the process can be optimized to not interfere with controlled stimulation. Another approach would be to bioprint stem cells without triggering differentiation, in order to fabricate immunotolerant and multipotent scaffolds. This review will discuss how bioprinting can be either uninvolved in stem cell differentiation, (i.e., bioinert) or can be included as part of process such that the components can act as stimuli.

The bioprinting techniques which are viewed as most suitable and widely used for cell delivery are ink jet bioprinting, laser bioprinting and extrusion bioprinting. The three techniques have different benefits and drawbacks for the delivery of stem cells, due to the differences in their mechanisms. Table 1 shows a comparison of the main features of the bioprinting techniques.

## 2. Extrusion Bioprinting

**Figure 2:**
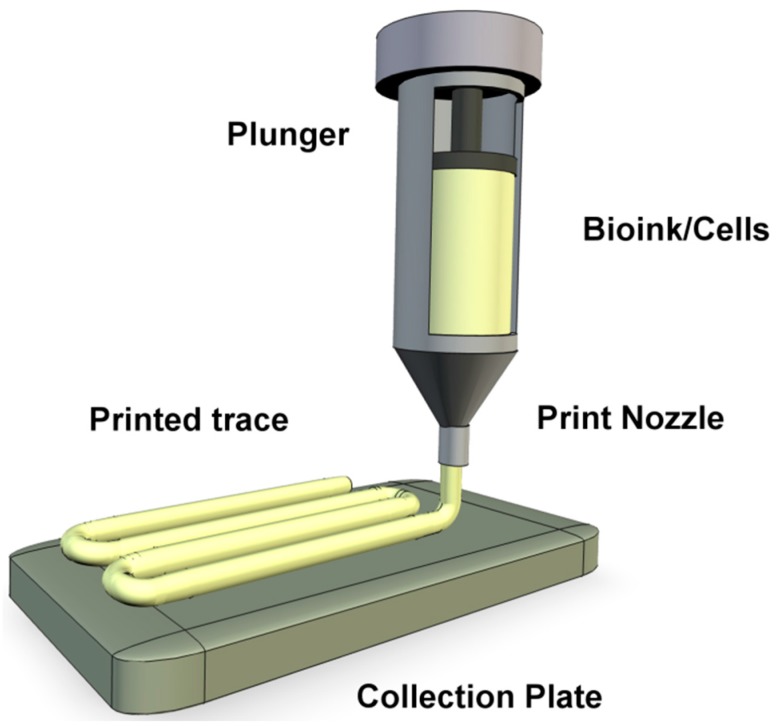
A representation of the extrusion bioprinting of stem cells. A stem cell bearing bioink is forced from a syringe reservoir and deposited as an extended trace on the collection plate.

Extrusion bioprinting (Figure 2) has been described as the most promising approach as it allows fabrication of organized constructs of clinically relevant sizes within a realistic time frame [9,10,11,12]. This technique produces a continuous bioink filament trace from a syringe, reminiscent to that of toothpaste emerging from a squeezed tube. The hydrogel bioink is forced from the syringe using either air (pneumatic) back pressure or mechanical screw plunger. The syringe is attached to print arm moving z–y direction over a collector moving the x-axis, so that the hydrogel can be deposited into 3D patterns [13,14,15] (this direction arrangement may vary between bioprinter models). Numerous biocompatible hydrogel polymers can be utilized as bioinks for extrusion bioprinting (choice for printing stem cells is discussed in Section 7: Bioinks) and has the ability to deliver a relatively high cell concentration. The back pressure apparatus is better for printing more viscous bioinks, however they suffer from a delay in control when attempting to halt bioink flow from the nozzle [12,15,16]. The mechanical screw plunger gives better control over the bioink flow conferring for improved patterning [12,15,16] however, for stem cell printing, mechanical screw extrusion can produce great pressure drops along the nozzle. The pressure drop is associated with the deformation and apoptosis of the bioink encapsulated cells [12,17,18].

## 3. Ink Jet Printing

**Figure 3:**
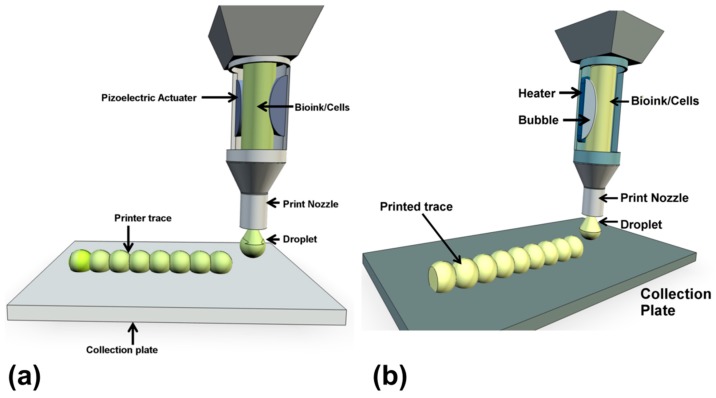
A representation of inkjet bioprinting of stem cell. Droplets of a stem cell bearing bioink are formed from the reservoir by the action of a piezoelectric (**a**) or thermal actuator (**b**) to be deposited on a collection plate.

Ink jet bioprinting was the first approach to be developed, from the modification of commercial ink jet printers [19] and as such resembles the mechanism of conventional ink jet printing of documents. Ink jet printing can be performed as continuous ink jet (CIJ) or drop on demand (DOD) [20]. The former produces an uninterrupted trace while the latter deposits discrete ink droplets on precisely coordinated positions, printing up in modular droplet units. Only the DOD approach is considered suitable for bioprinting since the CIJ method requires a conductive ink [20]. A cartridge is loaded with cells in hydrogel bioink and is printed in well placed droplets that are generated from a print head by the close control of a thermal or piezoelectric actuator (Figure 3) [21]. The thermal system is more prevalent for cell printing, even though the element temperature of the actuator can exceed 200 °C, the cells experience only minor, temporary temperature changes during the process. Currently, inkjet print heads tend to employ Micro-Electro Mechanical system (MEMS) that do struggle with printing highly viscous materials or high cell concentrations [21].

## 4. Laser Assisted Printing

**Figure 4:**
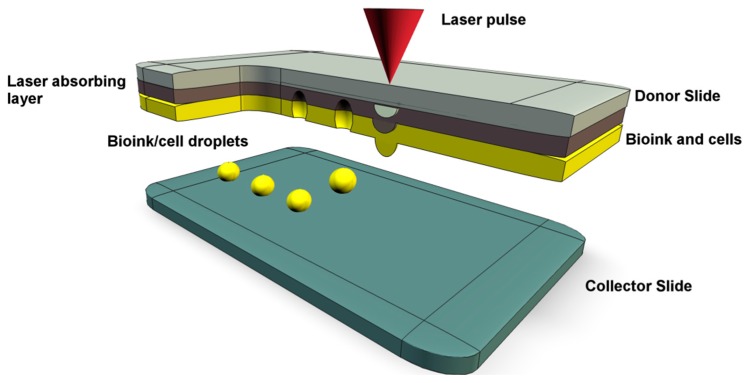
A representation of laser assisted bioprinting of stem cells. The laser absorbing layer is stimulated by the laser pulse to generate a pressure bubble, thus propelling the stem cells onto the collector slide.

This approach was developed from laser direct write and laser induced transfer technologies. The method functions by focused laser stimulation of the upper surface of an energy absorbing sacrificial layer. A cell containing biopolymer or bioink is coated onto the lower surface of the energy absorbing layer. Laser stimulation vaporizes the sacrificial material, generating a pressure bubble that propels a bioink droplet on to a collection/receiving substrate with precision placement (Figure 4) [19,22].

Unlike the inkjet printing, laser assisted bioprinting does not require a dispensing nozzle. This reduces the shear stress experienced by the stem cells during deposition [21] and also removes the occurrence of nozzle clogging [19].

This method is considered to have good cell printing properties, as it is able to use viscous bioinks and prints high cell densities with good post deposition viability. Moreover, the resolution can be as low as 10 μm [21]. However, the technique is considered the most expensive and complex [21].

## 5. Choice of Bioprinting Technique on Stem Cell Differentiation

No side by side studies have been performed to elucidate the comparative effects of these bioprinting techniques on printed cell stemness. However Lee et al. have discussed the comparative advantages and disadvantages of each process. Both shear and mechanical stresses have been observed to trigger differentiation in stem cells. Shear stress leads to differentiation to both endothelial and osteoblasts [22] and mechanical stress can stimulate chondrogenic and osteogenic differentiation [23]. This would indicate that laser assisted and inkjet bioprinting are superior to extrusion bioprinting due to the “gentler” processes involved. However, limiting oneself to these processes would in turn lead to the loss of the advantages of extrusion bioprinting stem cells, such as the printing of physiological cell densities and with high density/viscosity biomaterials [19]. It would therefore be advisable for researchers to monitor the status of the stem cells throughout the bioprinting procedure, especially for extrusion printing. The effect of shear stress on stem cells during extrusion bioprinting may be mitigated through the optimization of back pressure and the composition of the bioink with the inclusion of shear thinning polymers (See Section 7: Bioinks).

## 6. Stem Cells

Stem cells are defined as a possessing self-renewal and multilineage potential i.e., they can be stimulated to form one of several different types of functional cells. There are three principle stem cell types commonly used in bioengineering: mesenchymal stem cells, embryonic stem cells and induced pluripotent stem cells.

Mesenchymal stem cells (MSC) are also referred to as mesenchymal stromal cells or multipotent stromal cells, all conveniently sharing the same abbreviation. They are characteristically defined by their propensity of differentiating into osteoblasts, adipocytes or chondroblasts in vitro; however, they also have the ability to become other mesenchymal and non-mesenchymal cell types such as myocytes, tendocytes, ligament cells, smooth muscle cells, endothelial cells, cardiomyocytes, hepatocytes and neural cells [24,25,26,27,28].

MSCs were originally isolated from bone marrow but have since been located in many tissues such as liver, lung, teeth, muscle, adipose, and perinatal/extraembryonic-associated tissue (i.e., amniotic fluid/Wharton’s jelly, umbilical cord, placenta) [24,26,27,28]. The particular source of MSC often confers advantageous facets compared to other sources. For example, the concentration of stem cells harvested from adipose tissue is significantly greater to that of the other sources [29]. Perinatal stem cells have a great capacity for expansion, such as amniotic derived stem cells that can be cultured for over 300 cell cycles with a doubling time of 36 h [30,31,32]. In addition, perinatal stem cells have a broader range of multipotency than “adult” stem cells and, unlike embryonic stem cells and induced pluripotent cells, they do not show tumorgenicity in vitro [30,31,32].

Interestingly undifferentiated MSCs are immunotolerated, giving the option of allograft implantation for the secretion of transgene therapeutic factors, moreover, undifferentiated MSC have immunomodulatory properties that can potentially be exploited to reduce graft versus host symptoms and block graft rejection following transplantation [33,34,35].

Embryonic stem cells originate within the inner cell mass of a blastocyst and have the potential to differentiate into somatic cells of all three germ layers [36], hence a greater multipotency than adult stem cells. They do, however, suffer from several drawbacks. Their application is associated with ethical controversy, differentiation is not readily controlled and the cells can be immunogenic.

Induced pluripotent stem cells (iPSCs) offer an alternative to embryonic cells without the ethical and immunogenic drawbacks of ESCs. They are similar to embryonic stem cells in that they form embryoid bodies, have very similar gene expression profiles and impressive multipotency. They represent somatic cells that have been dedifferentiated into progenitor cells, Yamanaka et al. produced iPSC from fibroblasts through the introduction of four transgenes by retroviral transfection: *Oct 3/4, Sox2, Klf4, and c-Myc* [36]. More complex cocktails of proteins, peptides, chemicals and other factors have been developed for greater reprogramming control.

The clinical use of ESC and iPSCs are challenged by the risk of in vivo teratoma formation, the presence of which can interfere with their regenerative function. In iPSCs the teratoma formation has been associated with the presence of residual undifferentiated cells. The removal of these undifferentiated cells prior to implantation may improve the outcome [37,38]. The use of iPSCs is also associated with carcinoma generation, due to the genomic integration of a lenti virus. Safer versions and virus free iPSCs are being developed to make them a more realistic option for regenerative medicine [39].

## 7. Bioinks

Bioinks have to meet several key properties for their function. Their viscosity must be optimized to allow controllable, uninterrupted flow yet maintain the printed trace integrity while the bioink sets, through solvent evaporation or polymer cross-linking. For 3D bioprinting, the set bioink is required to hold the vertical print and bear the weight of the emerging structure. As the bioink is required to interact with cells in vitro and in vivo, the building material in the bioink is required to be cytocompatible. There is also a concern for any toxicity in the setting process, whether solvent evaporation or a molecule cross-linking process. Unfortunately the majority of biocompatible polymers that are able to form robust, vertically built up structures tend to be the ones requiring high temperatures and toxic solvents such as polycaprolactone, poly-l-lactide, poly(lactic-co-glycolic acid) etc. [40].

Cell printing bioinks have the further requirements; to maintain cell integrity and viability during resuspension and passage through the print head and provision of a suitable environment for cell growth and function within the printed scaffold. This limits aqueous materials to form bioinks, hence they tend to be soft hydrogels with high water content. Both natural and synthetic polymers are chosen [6,15,16,25,40,41,42,43,44,45,46,47,48,49,50,51,52,53,54,55,56]. Natural extracellular matrix (ECM) components have been used widely such as collagen, fibrin, gelatin, hyaluronic acid, etc. These bioinks provide a natural ECM like environment for the printed cells, especially collagen and its derivative gelatin. Other natural polymers include the polysaccharides chitosan and alginate. Synthetic biocompatible polymers such as pluronic F127, polyethylene oxide and polyethylene glycol are used. Table 2 displays the bioink properties, crosslinking features and application for 3D bioprinting of stem cells.

The rheological nature of the cell bearing bioink has a considerable impact on the cells, especially for the extrusion technique. The deposition of viscous polymers through a fine nozzle would impart an increasing shear stress as cells enter the nozzle. Indeed, using gelatin as a bioink, it was found that slight increases in gelatin concentration reduces the viability of the printed cells [14]. For most cell types, survival is a key consideration as regards the design bioprinting protocols.

Since the polymers forming bioinks are generally water soluble, cross-linking is required to form stable, long-lasting scaffolds. For example, classic cell encapsulation employs the water soluble alginate, it also represents approximately 25% of publications on bioinks [54]. Alginate is a polysaccharide composed of α-l-guluronic and β-d-mannuronic acid monomers, the ratio of which confers tunability to the subsequent hydrogel [62]. High mannuronic content produces a pliable gel whereas as guluronic content produces a stiff gel [62,63,64]. It requires a very straight forward cross-linking procedure, as the water soluble alginate forms a stable hydrogel following immersion in a solution containing multivalent ions such as calcium and barium [62,64]. This cross-linking involves the interaction of the alginates’ carboxylic acid groups with the multivalent ion, which occurs fairly rapidly, and at room temperature; therefore, the process can be triggered upon release from the print head nozzle and exposure to the multivalent ions. This makes alginate highly suitable for bioprinting [54,62,63,64].

Cell-bearing alginate can be printed directly into calcium containing solution (often calcium chloride). However, the polymer has low viscosity and is highly soluble, so immersion into the CaCl_2_ solution weakens the printed trace thus hampering 3D build up. There are several approaches to overcome this problem, such as: printing onto a CaCl_2_ gelatin substrate/solution [65,66], printing the CaCl_2_ solution onto the emerging scaffold [67], partial CaCl_2_ crosslinking of the bioink pre-printing, then gradual submersion of the emerging scaffold in CaCl_2_ [68], inclusion of gelatin into the alginate bioink to initiate gelation [69], using CaCl_2_ solution as the bioink printed into an alginate solution [70], the cell bearing alginate construct can be treated with a CaCl_2_ aerosol spray [71,72], and finally it can be bioprinted as a core shell, with stem cells in an alginate bioink as the core and CaCl_2_ solution forming a cross-linking outer shell [72].

For other bioinks cross-linking reagents such as glutaraldehyde, 1-ethyl-3-(3-dimethylaminopropyl) carbodiimide hydrochloride (EDC) and genipin may be employed [73]. However, these are not widely applied for bioprinting cells, since there is the potential for toxicity, especially for glutaraldehyde. Genipin, an extract from gardenia fruit, is viewed as the least cytotoxic and thus the most suitable for including in cell bearing bioinks. It is able to cross-link functional amine groups present on natural polymers and produces a more stable gel than commonly used carbodiimides such as EDC due to a longer crosslinking distance [74]. Genipin has been used to fabricate hydrogels used to support stem cell differentiation [75], however, due to the relative high cost of genipin and limited study, not much has been established on its effectiveness in cell bioprinting [73,76].

Enzymatic cross-linking also has been used for the protein-based hydrogel bioinks. In particular, mushroom tyrosinase and microbial transglutaminase. Cross-linking using such enzymes has the advantage of relatively mild reaction conditions for the encapsulated cells, i.e., neutral pH, aqueous medium and physiological temperatures [77]. Tyrosinase converts exposed tyrosyl side chains to *o*-quinones which are thought to react spontaneously with the side chains of lysine, tyrosine, histidine and cysteine residues [78,79], whereas transglutaminase catalysis a transamidation between glutamyl and lysyl side chains of proteins [78]. The latter cross-links at a slower rate than tyrosinase, however its activity results in a stronger hydrogel [78,80]. Tyrosinase generated *o*-quinone residues can also react with nucleophilic amines of non-protein polymers such as chitosan [81]. The use of enzymes in the bioink leads to a limiting printing time window before the viscosity clogs the print head [14]. Mammalian transglutaminase would overcome this problem as the enzyme is calcium activated, however, the current cost of this enzyme is prohibitive [78,82].

A favored method for bioink setting is by cross-linking a light curing polymer. There are several light activated cross-linking reagents, however, low-intensity, long-wave length ultra violet light activated photoinitiators [83,84] have proved to be useful for fabricating bioengineered hydrogels as they require low energy activation and relatively quick cross-linking times [55,83,84,85,86]. Upon exposure to UV, the photoinitiators produce reactive species such as free radicals [86]. The free radicals react with the vinyl groups on the monomers and in doing so, generating the covalent bonds that form the cross-linked hydrogel [70,83]. The UV irradiation and generation of free radicals have the potential to damage encapsulated cells, however, initiators such as 2-hydroxy-1-[4-(hydroxyethoxy)phenyl]-2-methyl 1-propanone (commercially known as Irgacure 2959) and lithium phenyl-2,4,6-trimethylbenzoylphosphinate (known as LAP) have been used for the cytocompatible cross-linking of cell bearing hydrogels. The photoinitiator concentration and UV irradiation intensity can be optimized for hydrogel properties and survival of encapsulated cells [55,83]. Indeed, excess photoexposure can interfere with MSC proliferation and cell cycle progression [87].

The UV initiated cross-linking via methacrylated groups have also been performed on other cell friendly polymers useful in 3D printing, such as Pluronic 127 PEG, polyvinyl alcohol, hyaluronic acid, dextran and chitosan to name a few [88]. In addition, unlike the chemical or enzyme crosslinking, the reaction is initiated upon deposition under a light source, making it ideal for bioprinting stem cells. The main drawback to the approach is the need to modify the hydrogel monomers to support the reaction.

It would be ideal for the crosslinker to act without influencing the stem cell phenotype, thus allowing lineage flexibility. During bioprinting the stem cells will be exposed to the crosslinking reactions that could conceivably affect stemness. The effect of crosslinking reagents on stem cell differentiation has mainly been examined in relation to the stem cells response to hydrogel stiffness (See Section 11.1: Bioink Stem Cell Microenvironment: Mechanical properties). Each crosslinker can generate a range of material properties since hydrogel stiffness is highly tunable through several variables such as the type of crosslinker, type of monomer, ratio of crosslinker to monomer, monomer concentration, type of initiator and initiator concentration [89].

Only genipin has been reported to have a direct effect on differentiation, stimulating odontogenic differentiation in human dental pulp cells (a source of MSC) [90]. This action occurs through the activation of extracellular signal-regulated kinase (ERK) signaling pathway by genipin. Although no studies have been performed on the direct effect of microbial transglutaminase on stem cell differentiation, it has been found that other transglutaminase isoforms do influence cell phenotype and behavior [91]. As stem cell bioprinting advances it will become important to fully elucidate the stimulatory effects triggered by crosslinking reagents.

## 8. Bioprinting with the Preservation of Stem Cell Multipotency

The bioprinting of undifferentiated stem cells may potentially allow the immunosuppressive properties of MSCs to be harnessed (as mentioned above) or for post-print differentiation by other factors. Indeed, a bioprinting method that does not have an inherent bias towards inducing a particular lineage can support greater variation in lineage commitment by relying on soluble factors as the stimulus (see Section 9 Differentiation of Bioprinted Stem Cells by Soluble Factors) [7]. As mentioned, the gentler, low shear techniques are less likely to trigger stimulation. For example, a laser bioprinting method has been demonstrated to deliver MSCs to an alginate/blood plasma hydrogel without any changes in viability, proliferation, apoptosis, or to stem cell phenotype when compared to unprinted cells [53].

In order to retain stem cell multipotency following extrusion or inkjet printing of cells, a bioink hydrogel with limited cellular interaction is beneficial, so as not to present a “target tissue” like environment to the stem cells that may promote differentiation.

Alginate, for example is one such bioinert hydrogel that allows the MSCs to be bioprinted whilst retaining their stemness. Blaeser et al. [92] studied the effects of shear stress on MSCs bioprinted in an alginate based bioink by a microvalve controlled back pressure assisted extrusion system. They found that shear stress forces endured during the print process had a great effect on the post-printing viability and proliferation rate yet no detectable effect on MSC phenotype (as demonstrated by the expression of MSC marker gene vimentin). The alginate printed cells managed to retain stemness, however, shear stress level had to be optimized to reduce cell death. They printed at a shear stress pressure of ≈5 kPa which conferred a printed cell viability of 94%. Interestingly the authors point out that although a print pressure of 5 kPa is considered low for bioprinting, it is profoundly greater than the 7 Pa encountered physiologically within the circulatory system [92]. 

The bioinertness may reduce the stem cells ability to proliferate and move within the printed gel, and may actually trigger cell death through anoikis (apoptosis induced by lack of cell attachment) [54]. For this reason, therefore, integrin binding peptide Arginyl-glycyl-aspartic acid (RGD) motifs have conjugated to alginate for inclusion in a bioink. These do aid the cells interaction with the printed bioink without necessarily triggering differentiation [93]. Moreover, adipose stem cell bioprinted in alginate lacking RGD have demonstrated increased integrin activity after eight days, indicating the cells may have the ability to secrete their own RGD containing ECM peptides [66]. 

Hyaluronic acid has been used for bioprinting stem cells and has great potential to produce a printed construct that retains multipotency. It is a non-sulfated glycosaminoglycan and forms a hydrogel that has already been used clinically [18]. Hyaluronic acid is an important and abundant constituent of the ECM of stem cell niches, such as that of bone marrow derived hematopoietic stem cells [94]. Unlike alginate, hyaluronic acid supports stem cell attachment through receptors such as CD44 [95], the interaction has the effect of maintaining stem cells in undifferentiated and proliferative states [94]. The interactions within a stem cell niche can also play an important role in supporting of differentiation route of the stem cell [96] and similarly, hyaluronic acid can be adapted to direct differentiation. For example, hyaluronic acid has been used in cardiogenesis, osteogenesis, chondrogenesis and neurogenesis of stem cells [94]. This polymer has, of yet, unrealized potential for the 3D printing and phenotype regulation of stem cells which hopefully will receive the future attention it deserves.

## 9. Differentiation of Bioprinted Stem Cells by Soluble Factors

In cases where the building materials do not influence the cells lineage fate, bioprinted stem cells can then be differentiated by incubation in medium containing soluble factors optimized to promote lineage commitment and maturation towards selected phenotypes. These soluble factors include growth factors and chemicals. 

Of the growth factors, TGF-β superfamily, Fibroblast growth factor (FGF), wnt signaling proteins, hedgehog proteins and notch 1 ligands are of considerable importance to stem cell fate. Transforming growth factor beta superfamily (TGF-β) of TGF-β and Bone Morphogenic Proteins (BMPs) are of particular interest to stem cell differentiation in vitro [97]. For example, TGF-β_1_ and BMP-6 are important for chondrogenic differentiation of MSCs [98,99]. When human ASCs seeded in 3D hydrogels fabricated from either alginate, agarose or gelatin were cultured in TGF-β_1_ containing chondrogenic media, the increased expression of cartilage matrix components (hydroxyproline and sulfated glycosaminoglycans) were detected [100,101]. Other widely used differentiation promoting growth factors include FGF, PDGF, IGF, EGF, and insulin, however, these factors tend to modulate the stimulation of the critical ones mentioned above [97].

Growth factors can also regulate the migration and behavior of stem cells. C17.2 neural stem cells were bioprinted alongside collagen and VEGF releasing fibrin hydrogels. It was observed that the cells had a propensity of migrating towards the VEGF loaded fibrin hydrogel and adopting a more differentiated like phenotype compared to the cells in VEGF free scaffolds. This study also demonstrates how a hydrogel bioink can act as a controlled release reservoir for growth factor [102]. 

Several chemicals have also been employed to control stem cell lineage. For example, combinations such as dexamethasone, ascorbic acid and β-glycerophosphate trigger MSC differentiation towards osteogenic or adipogenic lineage [103]. In another study, treatment with isobutylmethylxanthine, indomethacin and dexamethasone induced adipogenic stimulation whereas β-glycerolphosphate, insulin and dexamethasone stimulated osteogenic differentiation [104]. The peroxisome proliferator activated receptor γ (PPARγ) agonists rosiglitazone aids the adipogenic differentiation of MSCs [105,106]. Finally, the DNA demethylating agent 5-azacytidine can confer a cardiomyocyte-like phenotype in adult bone marrow MSCs [107]. 

Such soluble factors are used to create culture medium to direct in vitro differentiation, and can also be used in conjunction with bioprinting. Du et al. [52] bioprinted bone mesenchymal stem cells (BMSC) into a methacrylamide gelatin scaffold by mechanical extrusion. Following the print, the scaffolds incubated in osteogenic medium for 14 days demonstrated a considerable increase in the expression of osteogenic reporter genes, such as osteocalcin (OCN), bone sialoprotein and alkaline phosphatase, as compared to BMSCs kept in growth media.

Human iPSCs and ESCs bioprinted in RGD coupled alginate hydrogels were differentiated towards hepatocyte-like cells by incubation in appropriate medium over 17 days. The differentiation process can be initiated prior to bioprinting, as the cells could be bioprinted following 6 days differentiation treatment and then allowed to resume it for the remaining 11 days after printing. At the end of the treatment, the printed and non-printed stem cells displayed comparable commitment towards the hepatocytic lineage, hence, the bioprinting did not influence the cells’ fate [93].

In the case of MSC bioprinted by laser assisted technique (laser-induced forward transfer or LIFT) directly into the differentiation medium, the printed cells could be induced towards osteogenic or chondrogenic lineage by including the appropriate components [53]. The printed MSCs were differentiated over 3 weeks towards osteogenic phenotype in medium containing dexamethasone, β-glycerophosphate, insulin, transferrin, selenious acid, and, ascorbate-2-phosphate [53], or towards a chondrogenic phenotype in a culture medium containing TGF-β_3_, sodium pyruvate, dexamethasone, insulin, transferrin, selenious acid and ascorbate-2 phosphate [53]. Interestingly, using this technique, cells can also be predifferentiated for several days before bioprinting. In each case, following stimulation with the supplemented media, bioprinted MSCs underwent differentiation in a similar manner to unprinted cells [53].

## 10. Differentiation of Bioprinted Stem Cells with Bioink Additives

Rather than depending on extrinsic soluble factors, additional additives can be included into the bioink to aid MSC differentiation. In a very straight forward approach, hydroxyapatite (HA) has been included into an MSC bearing alginate-gelatin bioink for bone tissue engineering, which can be printed without adversely effecting cell viability or the printing [108]. The inclusion of HA enhances the osteogenic effect on printed stem cells [109,110] as observed with an alginate bioink containing osteoinductive biphasic calcium phosphate particles (BCPs), which consist of HA and β-tricalcium phosphate. Following in vivo implantation into a mouse model, these scaffolds were found to have substantially more evidence of osteogenic differentiation than scaffolds lacking BCP [109]. Similarly, another bioinert polymer PEG was used to create osteogenic bioinks. Poly (ethylene glycol) dimethacrylate (PEGDMA) bioinks were made containing either HA or bioactive glass as bone promoting additives. It was found that MSCs in PEG-HA bioink underwent greater osteogenic differentiation and increased collagen secretion for ECM remodeling [111].

Microcarriers (MCs) are polymer microspheres that provide a surface for cell adhesion and growth for attachment dependent cells in suspension [112,113,114,115]. They are produced in a wide range of sizes from 60 to 400 μm [112,113,115]. MCs have been found to support MSC adhesion and proliferation without affecting the cells multipotency [112]. Interestingly, MC can also be exploited for the modulation of stem cell differentiation within a hydrogel [114]. Firstly the size of the MC can be optimized to control the polymer stiffness, surface growth area on the particle and the cell organization [112,114]. These effects are not fully understood and the research on such is predominantly empirical in nature [114]. MCs, however, can be modified with bioactive molecule and releasable factors in order to trigger stem cell differentiation. Both human and murine MSCs undergo spontaneous osteogenesis when seeded on collagen coated polystyrene MCs [116]. Levato et al. functionalized the surface of polylactic acid MCs with recombinant collagen type 1 [112]. MSCs were preseeded on the MCs before suspension in a gelatin methacrylate-guar gum bioink and it was found that the MSCs had increased expression of OCN and deposition of mineralized matrix signifying osteogenic differentiation when compared with MSCs in bioink without MCs. The MC-MSC containing bioink could be printed by extrusion without clogging the nozzle or affecting the MSCs viability, and could generate a 3D structure with homogenous distribution of MC-MSC particles [112].

Pharmacologically active MCs have been applied to influence MSC differentiation, such as the fibronectin coated poly-lactic-co-glycolic acid (PLGA) MCs that release TGF-β_3_ in order to initiate chondrogenesis [117]. As regards bioprinting, gelatin spheres 75–125 μm in diameter (referred to as micropaticles) were fabricated to release BMP-2 in an alginate bioink to stimulate osteogenesis of goat MSCs. The printed scaffolds containing MCs showed significant osteogenic differentiation both in vitro and in vivo [118].

The bioink itself can be made pharmaceutically active. For example, BMP-2 attached to collagen microfibers were included into a gelatin methacrylamide bioink used for printing bone MSC [52]. The collagen microfibers were attached to the BMP-2 via a collagen binding domain. This facilitated the controllable release of the growth factor. The stem cells printed in this bioink and cultured in normal growth media demonstrated more enhanced osteogenesis than cells printed in bioink without collagen microfibers but cultured in osteogenic medium [52].

## 11. Bioink Stem Cell Microenvironment

Hydrogels can guide stem cell lineage commitment by mimicking natural ECM. In vivo, the ECM that surrounds the stem cells residence forms a niche that can modulate the cells’ survival, self-renewal, and proliferation. It positions the cells in contact with the required structural support, trophic support, topographical information and physiological cues for the stem cells to function in that particular environment [119]. In particular, the surrounding ECM mechanical properties and material composition play a critical role in stem cell fate and function [120]. The stem cells “communicate” with their surrounding environment through a family of ECM binding proteins in the cell membrane known as integrins. These proteins support inside-out and outside-in lines of communication between the cell and extracellular space [121]. Integrins are key to many important cellular events such as motility, viability, proliferation and differentiation [121]. Integrins were shown to be involved in stem differentiation when MSCs were seeded in RGD-modified hydrogels with and without α5-integrin and αV-integrin blocking antibodies. Although there was no change in cell viability with the inclusion of the blocking antibodies, it was found α5-integrin blockade reduced osteogenic differentiation whilst upregulating adipogenesis and αV-integrin inhibition noticeably decreased the rate of osteogenesis [121]. Different ECM components preferentially interact with certain integrin family members and individual cell types have the propensity to express characteristic range of integrins, hence there is great potential of various ECMs modulating diverse fates over several cell types.

### 11.1. Bioink Stem Cell Microenvironment: Mechanical Properties

The effect of ECM mechanical properties on stem cell differentiation is well established. In 2D cell seeding MSCs have been shown to differentiate towards a cell lineage compatible with the mechanical properties of the surface. As such MSCs adhering onto a relatively elastic substrate differentiate towards neuronal lineage (0.1–1 kPa), whereas the cells on a surface of intermediate stiffness differentiate towards skeletal muscle (8–17 kPa) and on a rigid surface the cells undergo osteogenesis (34 kPa) [120,122,123]. Similar effects have been observed for stem cells in 3D hydrogels. MSCs seeded inside a matrix of intermediate stiffness (11–30 kPa) became committed towards osteogenic lineage whilst the MSCs encapsulated in a softer hydrogel (2.5–5 kPa) tend towards adipogenesis [124]. This phenomena can be exploited in bioprinting by utilizing bioinks that produce the desired stiffness following gelation.

Gao et al. (2015) [41] combined PEG and GelMA bioinks for optimized bone and cartilage inkjet printing. They produced a bioprinted hydrogel with a compressive modulus of 1–2 MPa, around 100 fold greater than most previously reported natural hydrogels and was regarded suitable for hard tissue engineering. MSCs bioprinted in this hydrogel displayed dramatic differentiation towards chondrocytes and osteocytes [41].

It is noteworthy that the effect of substrate stiffness on cell differentiation may be dependent on the ECM components. For example, MSCs seeded onto polyacrylamide gels of high, medium and low elasticity differentiate towards neurocytes, myocytes, and osteocytes respectively when the gels were coated with collagen I or fibronectin, but not with laminin or collagen IV [122,125,126].

Interestingly, even different hydrogel cross-linking methods could alter the fate of printed MSCs in identical bioinks. Das et al. (2015) observed that MSCs cultured for 3 weeks in a bioprinted silk fibroin-gelatin hydrogels cross-linked with tyrosinase tended to show signs of differentiation towards chondrocytes [51]. However MSC similarly cultured with the exception of physical hydrogel cross-linking by sonification, appear to predominantly differentiate towards osteocytes [51].

### 11.2. Bioink Stem Cell Microenvironment: Materials

Natural ECM contains components that influence behavior and fate of the entrapped cells. This modulation tends to be tissue-specific, promoting cell function pertinent to that particular to that organ [127]. Tissue specific ECM can be decellularized though trypsinization and stringent washing (i.e., for the removal of all cellular and immunogenic material). The decellularization process removes all cells and cell debris while retaining the protein and glycosaminoglycan components [50]. The subsequent decellularized ECM (dECM) scaffold can be repopulated with cells from another source. Scaffolds fabricated from cardiac dECM have been shown to retain the ECM environmental cues suitable for hESCs differentiation to cardiac cells [50,128].

The dECM can be further disrupted and solubilized to produce a bioactive and tissue specific hydrogel [40,49,129]. This is often performed by treatment with pepsin and acetic acid to solubilize the collagen component, followed by lyophilization and grinding to powder [50]. Recently, there have been attempts to fashion bioinks in a similar manner, in particular, Pati et al. (2014) [50] produced dECM from adipose, cartilage and heart to produce bioinks to print tissue analogs. These dECM were used as bioinks to bioprint hASCs and human inferior turbinate-tissue derived MSCs. The cells could be deposited be by syringe extrusion with high viability of over 90%. In each case the cells expressed marker genes of their respective tissue at a significantly higher rate than cells printed in a collagen bioink, thus demonstrating the effectiveness of dECM as a bioink for promoting the differentiation of printed stem cells.

The drawback to using dECM is the complex processing required to remove all cellular and immunogenic debris without causing substantial loss or damage to the ECM components. It is also important to ensure that the harsh chemicals used are removed from the processed dECM.

Matrigel™ is a commercially available preparation of solubilized ECM proteins and glycosaminoglycans such as collagen IV, laminin, and perlacan derived from Engelbreth-Holm-Swarm mouse tumor [61,130]. Its composition confers a good ECM like environment, promotes osteogenic differentiation and its gelation properties (liquid at 4 °C and solid at 37 °C) makes it suitable for bioprinting. Unfortunately, Matrigel™ lacks batch to batch consistency, has a long gelation time and is not clinically approved for clinical application due its origin. For such reason it has not been widely employed for 3D printing [61].

## 12. Generation of Topographical Guidance Grooves and Controlled Stem Cell Deposition by Extrusion Bioprinting

The topographical guidance of cells on a biomaterial surface is known to influence the stem cells phenotype and cellular behavior [131,132]. For example, parallel surface grooves/channels can align cells while conferring an elongated morphology, which has been found to promote differentiation of stem cells towards several cell types including ligament fibroblasts, neuronal and cardiomyocytes [13,133,134,135,136]. We found that an extrusion bioprinter could firstly etch surface grooves and then deliver stem cells directly to these features in a two-stage procedure. Firstly the bioprinter was modified with the addition of an etching stylus in the place of the print nozzle. This enabled the bioprinter to etch a preprogrammed pattern into a hard polymer surface (e.g., polystyrene or polycaprolactone). Secondly, the printer was loaded with a 2% gelatin bioink containing suspended stem cells. The printer then used an identical programme as the first step to deliver stem cells to the etched grooves. The deposited bioink was not cross-linked, however it retained the integrity of the printed trace. The bioprinted stem cells managed to sediment to the polymer surface where they sensed and aligned along the etched grooves in discrete lines following the printed trace [13]. Cells seeded to the etched surface in culture media rather than bioink did show alignment, however, they grew evenly across the entire surface. MSCs bioprinted in this manner demonstrated marker gene expression associated with cardiomyocyte lineage commitment. In summary, this study identified an additional role for 2D bioprinting, the patterning of cell differentiating surface features, followed by the controlled placement of stem cells to the features [13].

## 13. Printed Stem Cell Differentiation as a Form of 4D Bioprinting

The concept of 4D printing is an extension of 2D and 3D printing in which the printed structure transforms over time in a predetermined manner post deposition [136,137,138]. In a review by Gao et al. 4D bioprinting is described as encompassing two particular concepts. The first involves printing with enhanced smart nanocomposites, stimuli-responsive shape memory alloys (SMA) and shape memory polymers (SMP) that transform or reshape post-printing in response to an external stimuli [137]. The second involves creating a construct which, post-printing, matures and evolves. This includes changes in cell functionality, cell organization, and alteration of the bioink composition. The actions performed by bioprinted stem cells conforms to the latter, through their lineage commitment, involving the formation of tissue structures such as vasculature, and secretion of ECM components following differentiation [136,137,138]. Indeed, the controlled differentiation of bioprinted stem cells can be viewed as a predetermined and time-dependent change within the scaffold. As described above, stem cell differentiation can dramatically alter the bioactivity and mechanical properties of the bioprinted construct [131], hence, the bioprinting and differentiation of stem cells may be considered as a form of 4D bioprinting.

## 14. Future Outlook

The great potential for the bioprinting/differentiation of stem cells will hopefully be realized for use in regenerative therapy, with an ultimate goal of fabricating customized tissue constructs and complete organs seeded with host stem cells for implantation. Some interesting in vivo studies have shown promising results for 3D printing for skin regeneration. MSC and AFS cells were directly extruded within a fibrin/collagen bioink over a full thickness skin wound, which demonstrated wound closure and re-epithelization greater than that of non-cellularized bioink control [25,31,139].

It has also been shown that printing stem cells alongside primary cells can improve functionality in vivo. Co-cultures of MSCs and endothelial cells (ECs) in a bioprinted scaffold can promote angiogenesis, potentially improving the accessibility of oxygen and nutrients to cells within large volume scaffolds. The effect was observed with cells delivered by LIFT on a polyurethane based heart patch construct. In this case, endothelial cells were printed in a grid shape and MSCs in square patterns within them. Subsequently, the heart patch was assessed in a rat heart infarction model and the scaffold with bioprinted cellularization demonstrated better arrangement of endothelial cells and improvement of cardiac function than the randomly cellularized one [140].

In another example, MSCs and Schwan cells were extruded layer by layer into cylindrical constructs alongside a supportive agarose hydrogel to form a bioprinted nerve graft that demonstrated an encouraging degree of regeneration in a rat sciatic nerve injury model. It was proposed that the stem cells could function by either secreting anti-inflammatory mediators or differentiating into Schwann cells [141,142,143]. There remains a need for therapeutics to treat spinal cord injury and, in particular, for successfully bridging injured nerve gaps. This is an ideal target for the bioprinting and differentiation of stem cells, in order to recover normal neural functionality [143].

One current flaw in bioprinting stem cells is the lack of mechanical strength imparted hydrogel bioinks. An interesting approach to overcome this is to combine the cytocompatible properties of bioink [136] with mechanical characteristics of hard polymers. The Atala group has used an “Integrated tissue-organ printer”, a pneumatic controlled printer which delivers hard polymers into the same scaffold as stem cell bearing hydrogels. The hard polymer imparts mechanical strength and structural integrity to the construct [144,145]. For example, in bone reconstruction, AFS cells were printed in a bioink comprising of gelatin, hyaluronic acid, fibrinogen and glycerol within a scaffold printed with polycaprolactone doped with tricalcium phosphate. Following the in vitro osteogenic differentiation of the stem cells, this construct performed well as a calvarial bone reconstruction in a rat model [145]. 

It is hoped that that bioprinting stem cells will eventually produce patient specific, bespoke constructs for regenerative therapy [7]. This may include matching the construct to a specific lesion size and shape, and even producing complete organs for implantation. It is recognized that this technology is still in its early days [54]. One particular challenge that needs to be overcome is the slow fabrication rate. It has been estimated with current bioprinting equipment, the fabrication of a full human liver (a construct of 1000 cm^3^) would take up to 3 days to complete. This lengthy production time may reduce the stem cell viability [7,54,57,58,144,146]. Another shortcoming facing the bioprinting of stem cells is the inability to deliver a concentration of cells that match the physiological packing in tissues such as the heart and liver. Starly and Shirwaiker noted that as current bioprinting techniques struggle to print concentrations up to 10^7^ cells/mL, hence improved technology will be required to meet the density of 5 to 10 × 10^8^ cells/mL they consider sufficient for tissue and organ function [20].

## 15. Summary

Bioprinting has the potential of generating tissue constructs for regenerative therapy and transplantation, furthermore, printing cellularized hydrogels can be used to create construct that biomimic natural soft tissues. 

Stem cells are an ideal candidate for seeding the constructs prior to implantation since they can be harvested in high numbers (adipose MSC in particular), expanded in vitro, and are immunosuppressive and multipotent. Stem cells are sensitive to external stimuli, hence the bioprinting process must not trigger the process in an uncoordinated manner. Although bioinks can be developed to be bioinert, they can also be designed to promote the desired lineage commitment. This can be done through soluble factors, bioink additives (growth factors and chemicals), material stiffness and polymer choice. Of notable interest is the use of dECM, which can be used to form a bioink containing cues for the promotion of stem cell differentiation towards the resident cell type of the tissue from which it was derived [50,96,128]. As mentioned, there is a shortage of donor tissue, however, decellularized collagen and major ECM components are highly conserved across species and are less threatened by rejection than allograft transplantation. The use of stem cells in tissue bioprinting has the important advantage when fabricating organs populated by non-proliferative cells, such as cardiac tissue, as stem cells can be expanded in vitro in order to accumulate sufficient numbers prior to differentiation. This has the potential of becoming more efficient as IPSC technology advances, as the cells required for the constructs can be obtained with minimal invasiveness, using dedifferentiated dermal fibroblasts [147].

## Figures and Tables

**Table 1:** A comparison of bioprinting methods. The table is a composite of bioprinting methodology comparison tables prepared from references [2,19,21,57,58,59].

Property	Bioprinting Methodology		
	Extrusion	Laser Assisted	Inkjet
Cost	Moderate	High	Low
Material Viscosity	High	Medium-high	Medium
Resolution	Medium (100 μm–1 mm)	High (≥10 μm)	Medium to High (10 μm–1 mm)
Print speed	Continuous/Slow	Medium/Fast	Fast
		200–1600 mm/s	1–10 k droplets/s
Fabrication time	Short	Long	Medium
Cell viability	Medium-High, 40%–95%	High, 95%	High, 85%
Cell density	High	Medium (<10^8^ cells/mL)	Low (<10^6^ cells/mL)
3D build up capability	Good	Medium	Low
Benefits for stem cell bioprinting	Prints ECM like hydrogels and cells at physiologically relevant density.	Low shear stress levels on cells during delivery, which may affect phenotype.	Nozzle free and exerts low shear stress on cells during deposition. High resolution.
Disadvantages for stem cell bioprinting	Prone to higher shear stress which can affect viability and phenotype.	Struggles with viscous polymers and high cell densities.	Can cause mechanical deformation of the cells.

**Table 2:** Biocompatible polymers used as bioinks for stem cell delivery are presented along with their crosslinking features and application in bioprinting stem cells.

Bioink	Properties	Crosslinking Features	Examples of Bioprinting of Stem Cells	Reference
**Alginate (Naturally derived polymer)**	Inexpensive, natural polysaccharide derived from algae. Bioinert, which may lead to anoikis and is often modified with RGD or additives such as hydroxyapatite. Crosslinking occurs rapidly hence alginate is very popular as a bionk.	Instant gelation in Ca^2+^ solution.	Fabrication of osteochondral tissue equivalents.	[6,44,46,53,54]
**Chitosan (Naturally derived polymer)**	A linear amino-polysacharride, soluble low pH, requires modification to be soluble at physiological conditions. Blended with gelatin for cell printing.	Crosslinked with gluteraldehyde when blended with gelatin.	No reports for printing with stem cells.	[54]
**Agarose (Naturally derived polymer)**	Bioinert. Forms cytocompatable and structurally stable hydrogels. Solidifies slowly, resulting in bioink spreading. Not biodegradable in mammals.	Thermal gelation, cells mixed at 40 °C and gelates at 32 °C. No other polymerizers needed.	Printing of bone marrow stromal cells in agarose has been assessed.	[6,16,43]
**Hyaluronic-MA (Naturally derived polymer)**	A non-sulfated glycosaminoglycan, usually used for producing soft tissue like hydrogels rather than ones confering structural stability. Often mixed with gelatin, dextran or other polymers to overcome bioinertness and mechanical weakness.	UV triggered free radical polymerization.	Adipose stem cells printed in Gel Ma/HA Ma hydrogel, confering high cell viability detected after 1 week (97%).	[25,40,45]
**Fibrin (Naturally derived polymer)**	Natural protein comprised of cross-linked fibrinogen, has quick crosslinking rate and is glue like in form. The mechanical stiffness is low, so often used in conjunction with other polymers.	Crosslinks through the thrombin cleavage of fibrin.	Blended with collagen to deliver stem cells by inkjet with the application of skin regenraion.	[25,54]
**Silk fibroin (Naturally derived polymer)**	Good biocompatability and mechanical properties. Mixed with gelatin to prevent nozzle clogging.	crosslinked with tyrosinase or by sonification.	Silk fibroin-gelatin bioink used to print human nasal inferior turbinate tissue derived MSC that supports multi lineage differentiation.	[51,54]
**Gelatin (Naturally derived polymer)**	Formed from partially hydrolysed collagen. More soluble than collagen. Melt/gelation temperature 30 °C–35 °C, requires secondary crosslinking for applications at physiological temperatures. Matrix can be remodelled by cells.	Crosslinked using gluteraldehyde, carbodimiide or transglutaminase. UV irradiation of the methycrylated form.	BMSCs printed in gelatin MA with BMP2 or osteogenic medium.	[40,52]
**Collagen type 1 (Naturally derived polymer)**	Rich in the intergrin binding RGD motif. The ionic or pH changes involved in gelation are usually not gentle enough to allow cell bioprinting, however water soluble forms do exist. Collagen hydrogels are formed at low concentration (<3 mg/mL) that confer for low elastic modulus. Unfortunately a 100% collagen hydrogel may not be ideal as a cellularized construct due to water exclusion and contraction induced by hydrophobic peptide reisdues.	Gels through hydrophobic bonding with a slow rate of crosslinking, so can be blended with faster crosslinking polymers such as alginate or fibrin.	MSCs in collagen hydrogel differentiate towards chondrocytes, expressing cartilage proteins.	[25,40,48,54,60]
**Decellularized ECM (Naturally derived polymer)**	Supplies a natural like ECM niche for the stem cells. The stem cells seeded in dECM scaffold show greater degree of differentiaiton than cells seeded in collagen.	Can form a bioink that remains as a solution below 15 °C and gels after 30 min at 37 °C.	Adipose, cartilage, and heart dECM used as cell printing bioink for adipose derived SCs and human inferior turbinate tissue derived MSC.	[49,50]
**Matrigel (Naturally derived polymer)**	ECM like hydrogel rich in laminin, collagen and heparan sulfated proteoglycan. Has been used extensively for 3D cell culture.	Thermal gelation.	Not widely employed for bioprinting, used for printing HepG2 cells by temperature comtrolled syringe.	[42,61]
**Methylcellulose (Naturally derived polymer)**	Can be used to aid printing of another polymer and is then released. Enhances print viscosity and porosity following release.	Thermal gelation.	Blended with alginate to print MSCs into a low concentration alginate hydrogel.	[43,46]
**Poly(ethylene glycol)/poly (ethylene oxide) (Synthetic polymer)**	Bioinert, variable molecular weight allows tunable properties, altering stiffness can aid stem cell differentiation. Can be easily conjoined to other molecules. Requires modification to allow crosslinking.	UV initiated photocrosslinking of the PEGDMA.	Bone marrow derived MSCs printed for osteogenic and chronogentic differentiation.	[15,41,47,54]
**Pluronic F127 (Synthetic polymer)**	Inverse thermogelling polymer. Bioinert and has poor cell spreading and reduced viability after printing.	UV crosslinkable diacrylate form (with LAP initiation) confers additional stability of printed scaffold.	MSCs printed in photo cross linked bioink were cultured in osteogenic medium.	[43,55,56]
